# Swedish-Norwegian co-operation in the treatment of three hypothermia victims: a case report

**DOI:** 10.1186/s13049-017-0418-5

**Published:** 2017-07-17

**Authors:** Anders Wetting Carlsen, Nils K. Skjaervold, Nils Johan Berg, Øystein Karlsen, Eli Gunnarson, Alexander Wahba

**Affiliations:** 1 Department of Cardiothoracic Anesthesiology and Intensive Care, St. Olavs Hospital, Trondheim University Hospital, Trondheim, Norway; 2 Department of Cardiothoracic Surgery, St. Olavs Hospital, Trondheim University Hospital, Trondheim, Norway; 3 Department of Circulation and Medical Imaging, Faculty of Medicine, NTNU, Norwegian University of Science and Technology, Trondheim, Norway; 4 Department of Emergency Medicine and Pre-Hospital Services, St. Olavs Hospital, Trondheim University Hospital, Trondheim, Norway; 5 Neuropediatric Unit, Astrid Lindgren Children’s Hospital, Department of Women’s and Children’s Health, Karolinska University Hospital, Stockholm, Sweden

**Keywords:** Accidental hypothermia, Resuscitation, Extracorporeal membrane oxygenation, Extracorporeal life support, Cardiopulmonary bypass, Extracorporeal circulation, Emergency medicine

## Abstract

**Background:**

Accidental hypothermia with cardiac arrest represents a challenge for pre-hospital rescuers as well as in-hospital staff. For pre-hospital personnel, the main focus is to get the patient to the correct destination without unnecessary delay. For in-hospital personnel early information is vital to assess the possibility for resuscitation with extracorporeal re-warming. The challenge is augmented when rescuers must cross national borders to reach and/or deliver the patients. We present a case where three adolescent boys suffered severe hypothermia after a canoeing accident in Sweden.

**Case presentation:**

Three 14-year-old boys were canoeing a mountain lake close to the Norwegian border when their boat capsized and they all fell into the cold water. The rescue operation was hampered by rough weather conditions, and immersion times spanned from 63 to 125 min. Flight times from the scene of accident to the nearest ECMO center in Norway (Trondheim) and Sweden (Umeå) were about 30 and 90 min respectively. Two of the victims showed no vital signs after retrieval from the water and had extremely low body temperatures. They were brought to Trondheim University Hospital where they were resuscitated successfully with extracorporeal re-warming. Unable to be weaned from ECMO in the initial phase, both patients were retrieved by mobile ECMO teams to Karolinska University Hospital, from where they were discharged to their homes with good outcomes, although with some sequelae. A third victim with moderate to severe hypothermia without cardiac arrest was treated at a local hospital, from where he after a short stay was discharged without physical sequelae.

**Conclusion:**

These cases are a reminder of the traditional mantra that «no one is dead until warm and dead». Good communication between pre- and in-hospital staff can be vital for optimizing patient treatment when handling victims of severe hypothermia, and especially when there is multiple victims. Communication between neighboring countries, but even neighboring regions within the same country, can be challenging. We encourage regions similar to ours to review protocols regarding hypothermia management, making them more robust before incidents like this take place.

## Background

Re-warming with the use of Extracorporeal Circulation (ECC), i.e. Cardiopulmonary Bypass (CPB) or Extracorporeal Membrane Oxygenation (ECMO), after severe accidental hypothermia with concomitant circulatory arrest is an established form of treatment and can be performed in all university hospitals in Scandinavia. A prerequisite for successful treatment is that the circulation of the victim has ceased due to hypothermia, and not anoxia. The brain should be well saturated and cooled before the heart stops. Resuscitation of these patients on site involves manual/mechanical chest compressions and intubation/ventilation before swift transportation to a hospital with ECC readiness. If the patient meets the criteria for ECC re-warming (i.e. core temperature < 32 °C and s-Potassium <8 mmol/l), the patient should be slowly re-warmed on CPB/ECMO according to local protocol, with the goal of re-establishing spontaneous circulation [1–3]. In the aftermath of such a maneuver, several and serious complications may be anticipated, such as respiratory failure, circulatory failure, renal failure, coagulopathies, limb ischemia, central and peripheral neural injury, etc. [4, 5].

Pre-hospital care in these cases is always challenging and correct real-time information is often sparse. Lack of information represents a substantial challenge also for the in-hospital crew, as details of the accident timeline are vital in order to assess the potential for extracorporeal re-warming [6]. Co-operation within the rescue chain is essential, as was co-operation and co-ordination across national borders in this case.

**Fig. 1:**
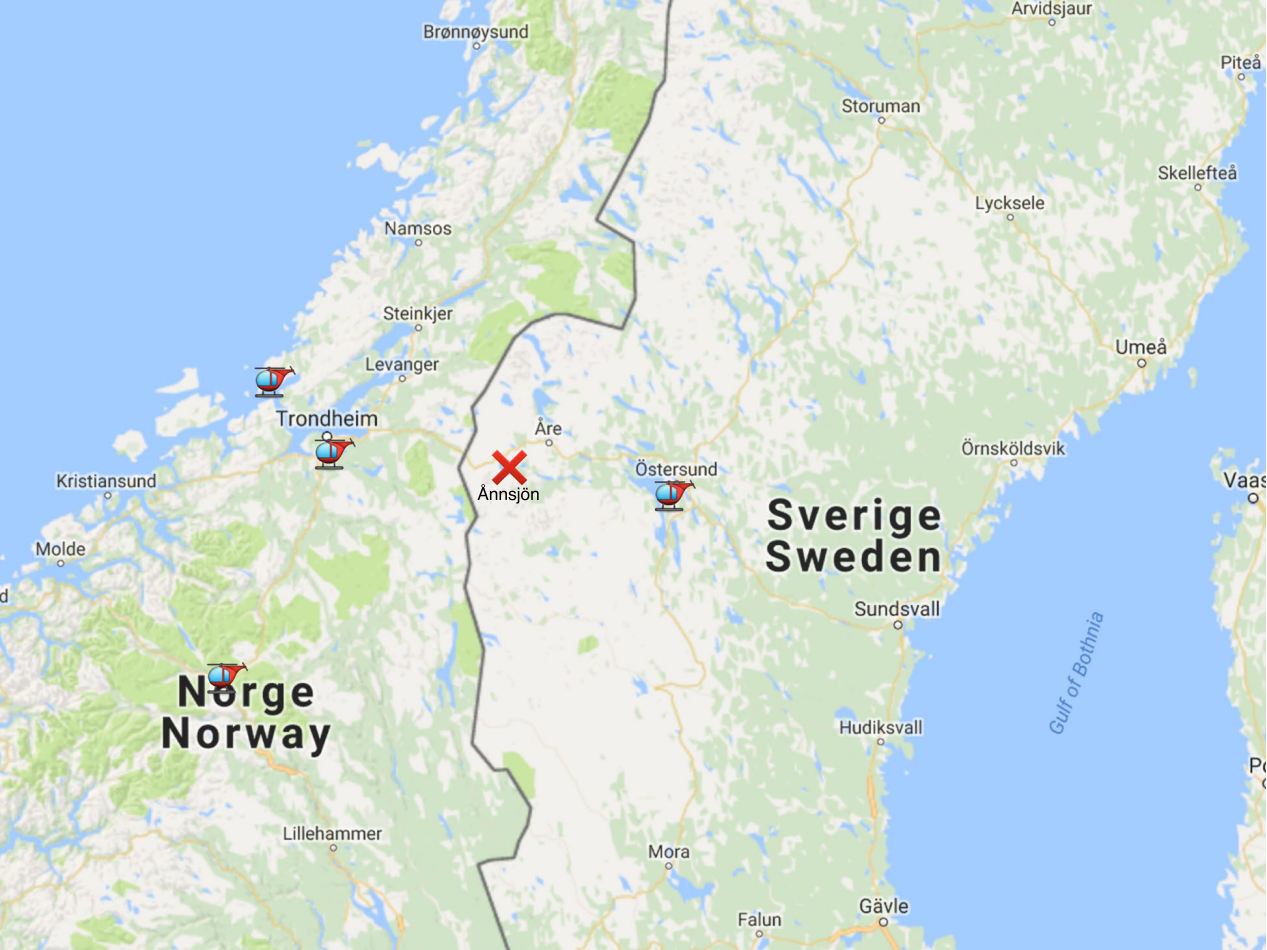
Overview map showing the scene of accident, ambulance and SAR helicopter bases and the geographic relation to hospitals with ECC capability (Trondheim (Norway) and Umeå (Sweden))

We present a case where two Swedish hypothermia victims of a canoeing accident in Jämtland, Sweden, were brought to Trondheim, Norway, for in-hospital resuscitation, this being the closest facility with ECC capability (Fig. 1). A third victim was cared for at a local hospital. After successful re-warming and initial intensive care treatment, both patients were transported to Karolinska University Hospital in Stockholm for further treatment.

## Case presentation

Three 14-year-old boys attending a summer camp in rural Sweden, were canoeing the mountain lake of Ånn (Ånnsjön), when their canoe capsized and they all fell into the cold water. The accident took place in a sparsely populated area in the county of Jämtland, between Åre and the Norwegian border. In spite of it being Mid-June, the weather in the area was poor, with strong gusts of wind and air temperature of 8 °C. The water temperature was 3-4 °C. The boys were all slim with no excessive body fat, and they were all wearing jeans, fleece jackets and life vests. Bystanders on shore observed the accident at approximately 16:40 and called it in at 16:44. The initial distress call was vague, but reported “people in the water”. An ambulance helicopter from Östersund, currently on another mission, was immediately re-routed towards Ånnsjön along with several local ambulances and a local mountain rescue crew.

### Patient 1

*Patient 1 was picked up from the water at 17:43, after being found floating with his head fully submersed. Basic CPR was started immediately in the rescue boat. Advanced CPR was started at 17:57 by the Östersund HEMS-crew as the patient was brought to shore. The patient was intubated, and manual chest-compressions were continued. An electrocardiogram initially showed asystole, with conversion to ventricular fibrillation after a few minutes. Helicopter transportation toward Trondheim was started after 25 min on scene. During transportation adrenaline 7 mg and amiodarone 300 mg was administered intravenously. He received 1200 ml of warm Ringer-Acetate and a total of 13 defibrillation attempts were made without return of spontaneous circulation.*

*The patient was admitted to St.Olavs University Hospital (Trondheim, Norway) at 19:05 under on-going resuscitation. Blood gas analysis (α stat) in the ER showed pH 6.48, Base Excess − 31, s-Potassium 4.8 mmol/l and s-Lactate 20 mmol/l. The nasopharyngeal temperature on admission was 18.0 °C and ECG showed ventricular fibrillation. The patient was transferred to the OR for re-warming with Cardiopulmonary Bypass (CPB). He was cannulated in the right femoral artery and vein and was slowly rewarmed to 36 °C. At 30 °C he was defibrillated successfully to sinus rhythm. Weaning the patient from ECC was impossible because of excessive pulmonary edema, and he was switched to veno-arterial Extracorporeal Membrane Oxygenation (VA-ECMO) and transferred to the ICU.*

*The next day the patient was still sedated and intubated. He was circulatory stable while on ECMO, and was brought “home” by an ECMO retrieval team from Karolinska University Hospital, Stockholm. He was successfully weaned from VA-ECMO after three days and extubated after seven days. Because of rhabdomyolysis and transient renal failure he received renal replacement therapy (RRT) for eight days. He was discharged from the ICU eleven days after the accident. He went through extensive rehabilitation at the children’s ward at Karolinska University Hospital and was discharged to his home 25 days after the accident. 18 months after the accident he has a remaining peripheral neuropathy, most dominantly affecting fine motor skills in his hands. He has regained a good ability to walk. He is cognitively intact, is functioning well psychologically, and is currently attending high-school. Magnetic resonance imaging (MRI) of the brain shows no pathology.*

### Patient 2

*Patient 2 had at an early point managed to re-enter the canoe and was waving to the rescuers as they were picking up Patient 1 at approximately 17:45. When the rescuers got back to him about 20 min later he was lying unconscious in the water, but still breathing. He reacted with some sort of spasms when being retrieved from the water and was brought to shore at 18:10 where first aiders provided him with dry clothes. At 18:46 he was transported by ambulance to the nearest hospital in the town of Östersund. Tympanic membrane temperature measured in the ambulance was 28.6 °C. ECG showed intermittent atrial fibrillation. During transportation he regained consciousness. He was warmed slowly using non-invasive methods, and was hemodynamically stabile with stable sinus rhythm after admission at 19:39. His lowest registered core temperature (bladder) was 29.9 °C. Blood gas analysis (α stat) at 20:10 showed pH 7.28, Base Excess − 5.7, s-Potassium 4.3 mmol/l and s-Lactate 1.2 mmol/l. He was discharged to his home without any physical sequelae after a short hospital stay.*

In parallel to the resuscitation of Patient 1 and 2, a search operation for the third victim of the accident took place. At 17:59 the Emergency Call Center in Trondheim received a request for assistance from rescue coordinators in Östersund. At 18:17 an ambulance helicopter with rescue divers took off from Trondheim heading towards Ånnsjön.

### Patient 3

*When the Trondheim HEMS-crew arrived at Ånnsjön at 18:45, Patient 3 had just been taken to shore. He too had been found lifeless in the water with his head fully submersed. Advanced CPR was started immediately on scene. Tracheal intubation was difficult, but was successful on third attempt with the use of a gum elastic bougie. Mechanical chest-compressions were established* (LUCAS2®, Jolife AB, Lund, Sweden)*, and continued throughout transportation. Initial ECG showed asystole. No medication was administered and no defibrillation was attempted during transport. Due to long distance to the nearest Swedish hospital with ECMO capacity (Umeå), Patient 3 was also flown to Trondheim, where he arrived at 19:45. A second ECMO re-warming team had been scrambled* ad-hoc *and stood ready on arrival.*

*On admission the patient was brought directly to the OR. Nasopharyngeal temperature was 14.5 °C. Blood gas analysis showed pH 6.56, Base Excess − 26.5, s-Potassium 5.2 mmol/l and s-Lactate 22 mmol/l. ECG showed ventricular fibrillation. VA-ECMO was established with vascular access in the right femoral artery and vein, and rewarming was started. At 25 °C the patient showed signs of waking up and required sedation. At 31 °C he was defibrillated into sinus rhythm. Due to pulmonary edema VA-ECMO was continued after reaching the goal temperature of 36 °C, and the patient was transferred to the ICU. During the following night the patient developed signs of compartment syndrome in his left forearm and right leg and a fasciotomy was performed in both locations. In spite of this circulation to the right lower limb was critically impaired. Exploration of the cannulation site showed that the distal bypass cannula was misplaced in the femoral vein, and after relocating it to the femoral artery circulation to the leg was greatly improved.*

*After having dealt with the leg ischemia the patient required additional fluid therapy, but was otherwise hemodynamically stable on VA-ECMO, and later the following evening he too was brought to Stockholm by an ECMO retrieval team from Karolinska University Hospital.*

*In Stockholm, he was weaned from VA-ECMO after a total of five days and extubated after twelve days. He went through several surgical procedures because of compartment syndrome in both arms and the right leg. He had transient renal failure due to rhabdomyolysis and received RRT for a total of 18 days. He was discharged from the ICU 19 days after the accident.*

*The patient went through extensive rehabilitation at the children’s ward at Karolinska University Hospital before being discharged to his home 2 months after the accident. 18 months after the accident he has peripheral neuropathy with general muscular weakness, impaired fine motor skills, and a limited, but steadily improving walking distance. He is affected by chronic pain (algoneurodystrophy) in his right lower limb. Brain MRI has shown ischemic lesions in his right temporal lobe which are consonant with his moderately impaired short-term memory and learning ability. Considering the graveness of his condition the overall outcome is absolutely positive. He still has potential for further improvement* (Table 1).

**Table 1:** Patient characteristics (all time measures in min)

	Patient 1	Patient 2	Patient 3
Sex, age(y)	Male, 14	Male, 14	Male, 14
Immersion time	63	90, partial	125
Submersion time	Unknown	-	Unknown
Initial registered rhythm	Asystole	AF/SR	Asystole
Chest compressions	Manual	No	Mechanical
Prehospital defibrillation	13 times	No	No
Prehospital Adrenalin administered	7 mg	No	No
HLR-to-ECC time	120	-	83
Lowest registered temp.	18.0 °C	28.6 °C	14.5 °C
s-Potassium on admission	4.8 mmol/l	4.3 mmol/l	5.3 mmol/l
pH on admission (α stat)	6.48	7.28	6.56

## Discussion and conclusion

### The Swedish-Norwegian co-operation

The first phase of a rescue operation like this is almost always chaotic and information tends to be deficient. The initial distress call was vague, and circumstances and the number of missing persons were unknown. The accident took place in an area far from professional rescue personnel. Although local rescuers did a formidable job on scene, one can in retrospect point out that more advanced resources should have been routed towards the scene of accident in an earlier phase. A request for Norwegian assistance was made about 1 h 20 m after the accident. With the wind conditions that day three helicopters from Norway (one SAR helicopter and two ambulance helicopters), each manned with an anesthesiologist and capacity to carry rescue divers, could have been on scene within 1 h. With sufficient resources patient 2 could have been transported to a hospital with ECMO competence in case his condition deteriorated. Earlier request for Norwegian assistance could have brought patient 1 and 3 to hospital earlier.

On scene, the Östersund HEMS-crew was confronted with an overwhelming scenario with multiple hypothermia victims, but realistically without capacity to handle more than one patient. Prior to this accident there existed no protocols, neither for pre-hospital workers nor for emergency call dispatch centers, allowing hypothermia victims requiring extracorporeal rewarming (ECR) to be admitted to a nearer ECMO facility across the national border. However, medical co-operation between Sweden and Norway has historically been good in our region, and ambulance helicopters sometimes even operate on the “wrong” side of the border. Faced with a lifeless young patient with profound hypothermia (grade IV of IV according to the Swiss hypothermia classification) [1] and an additional 60 min flight time to the nearest ECR-center in Sweden, the HEMS-crew decided wisely to transport Patient 1 to Trondheim. Necessary clearances were handled quickly between the Swedish and Norwegian emergency call centers. Direct radio communication between helicopter and hospital/ECR-team was technically impossible at the time. Even though Scandinavian languages are quite similar, a language barrier led the ECR-team to think they were about to receive a couple of two-year-olds (the Swedish word for “teenager” had been confused with the Norwegian word for “two-year-old”). This misunderstanding was sorted out mere minutes before the arrival of Patient 1.

According to the Swiss hypothermia classification, Patient 2 had clinically stage II/III hypothermia (unconscious/impaired consciousness) when he was picked up from the water, indicating moderate to severe hypothermia. The pre-hospital course has not been available in full detail, but at least his sensorium was affected and he had intermittent atrial fibrillation indicating a possible circulatory impact of the hypothermia. Guidelines for management of accidental hypothermia advice that patients with moderate and severe hypothermia (Swiss hypothermia classification, stage II and III) with concomitant unstable circulation (systolic blood pressure < 90 mmHg, ventricular arrhythmias or core temperature < 28 °C) should be transported directly to an ECMO/CPB center. Whether or not Patient 2 should have been brought to a hospital with ECR capability is debatable. Ideally, we think he probably should have, based on initial clinical signs, time of exposure in the cold waters, likelihood of temperature afterdrop, and last but not least the distance to the nearest ECR-center with capacity (Umeå, Sweden) had his condition deteriorated. Core temperature is often difficult to obtain in a pre-hospital setting. This said, we acknowledge that there were insufficient resources on scene, and we agree with the priorities made to move him by ambulance to the nearest hospital.

Patient 3 also had Swiss grade IV, severe and profound hypothermia. The decision to bring him to Trondheim University Hospital was probably psychologically easier for the pre-hospital crew, Trondheim being home base for the ambulance helicopter, but it underlines the importance of adequate communication between pre-hospital and in-hospital services. ECR is a treatment demanding large resources, both personnel and equipment, and is in Scandinavia available only in cardiothoracic surgical centers. In Norway, the cardiothoracic units are small, and the capacity for receiving patients in need of ECR is variable, even within the same institution, and depends on available personnel, number of available ECMO/CPB machines, and ICU capacity. A higher number of victims in need of ECR in this case would probably have exceeded our capacity, and would have demanded patients being routed to other destinations.

Studying this rescue operation one does find room for improvement, especially when it comes to communication, but it has also shown that good co-operation across national borders can produce excellent treatment results. In the aftermath of this accident routines have been reviewed by a Swedish-Norwegian regional meeting, and protocols were created to make similar incidences in the future run more smoothly. Communication being the key word, it mostly boils down to optimizing lines of communication and finding strategies to improve communication from pre-hospital to in-hospital staff and vice versa. Ongoing implementation of digital radio communications in both countries and harmonizing radio frequencies will hopefully also be a powerful tool to this end.

The ECMO center at Karolinska University Hospital was contacted on the morning after the accident. Being the largest ECMO center in Scandinavia, we turned to them for discussion on when transfer of the patients would be practicle. Given their clinical state, and since the patients required all of our ECMO capacity, we agreed that the patients be retrieved on ECMO the same day. In retrospect we find this was the best solution for all parties: The patients were treated further in a highly experienced and specialized hospital. The patients were brought closer to home and their next of kin. The ECMO capacity of our hospital was restored in case of new emergencies.

### The choice of resuscitation strategies

Pre-hospital treatment of Patient 1 and 3 differed in that Patient 1 received multiple defibrillation attempts and adrenaline/amiodarone administered according to standard normothermic ACLS algorithm, whereas no defibrillation attempt or medication was administered to Patient 3. The use of medication is debated, and little evidence about its use exists. Resuscitation guidelines recommend withholding adrenaline, other CPR drugs and shocks until the patient has been warmed to a core temperature ≥ 30 °C [2]. In both patients wet clothes were removed in order to prevent further heat loss. In our opinion the differences in pre-hospital treatment had no obvious effect on outcome in this case. Patient 3 received mechanical chest compressions whereas Patient 1 received manual compressions. The choice between the two is probably based on personal preferences and available equipment, and as long as compressions are performed uninterruptedly and with adequate quality, this should not be crucial for outcome [7].

### The choice of re-warming strategies and important pitfalls

In-hospital treatment also differed between the patients. Firstly, both patients fit the criteria for considering ECR (core temperature < 32 °C and s-Potassium <8 mmol/l). Patient 1 was re-warmed on CPB and later switched to ECMO while patient 3 was re-warmed directly on ECMO. The choice was basically based on the surgeon’s preference, as each of the methods have their pros and cons. In our experience with accidental hypothermia involving submersion, post-ROSC pulmonary edema and/or transient cardiac failure are significant complications which often require additional treatment on ECMO. One could therefor argue that ECMO should be first choice in rewarming patients with hypothermic circulatory arrest [3].

In Patient 3, the bypass cannula to the lower limb was initially inserted through the femoral artery into the femoral vein. In spite of surgical cut down in the groin and skilled surgeons, correct insertion of the cannulas is difficult during ongoing resuscitation. Malposition of the ECMO cannulas may lead to increased mortality and morbidity. Assessment of cannula position with ultrasound may decrease risk of malposition [8]. Many ECMO centers trend toward minimally invasive, ultrasound guided percutaneous insertion of cannulas.

## Abbreviations

ACLS: Advanced Cardiovascular Life Support
CPB: Cardiopulmonary Bypass
CPR: Cardiopulmonary resuscitation
ECC: Extracorporeal Circulation
ECG: Electrocardiogram
ECLS: Extracorporeal Life Support
ECMO: Extracorporeal Membrane Oxygenation
ECR: Extracorporeal Re-warming
ICU: Intensive Care Unit
MRI: Magnetic Resonance Imaging
ROSC: Return of Spontaneous Circulation
SAR Helicopter: Search and Rescue Helicopter
VA-ECMO: Veno-Arterial ECMO
